# Antimicrobial Policies in United States Beef Production: Choosing the Right Instruments to Reduce Antimicrobial Use and Resistance Under Structural and Market Constraints

**DOI:** 10.3389/fvets.2019.00245

**Published:** 2019-07-19

**Authors:** Guillaume Lhermie, Leslie Verteramo Chiu, Karun Kaniyamattam, Loren William Tauer, Harvey Morgan Scott, Yrjö Tapio Gröhn

**Affiliations:** ^1^Department of Population Medicine and Diagnostic Sciences, College of Veterinary Medicine, Cornell University, Ithaca, NY, United States; ^2^Dyson School of Applied Economics and Management, Cornell SC Johnson College of Business, Cornell University, Ithaca, NY, United States; ^3^Department of Veterinary Pathobiology, College of Veterinary Medicine and Biomedical Sciences, Texas A&M University, College Station, TX, United States

**Keywords:** policy analysis, antimicrobial use, antimicrobial resistance, beef production system, policy instruments, economics

## Abstract

Antimicrobial use (AMU) in animal agriculture contributes to the selection of resistant bacteria, potentially constituting a public health threat. To address antimicrobial resistance, public policies set by governments, as well as intra-sectoral approaches, can be implemented. In this paper, we explore how common policy instruments such as regulations, economic incentives, and voluntary agreements could help reduce AMU in beef production. We first describe the structure of the beef supply chain which directly influences the choice of policy instruments. We describe how externalities and imperfect information affect this system. We then discuss how five policy instruments would each perform to achieve a reduction in AMU. Bovine respiratory disease complex (BRD) represents the major driver of AMU in beef production; consequently, reducing its incidence would decrease significantly the amounts of antimicrobials administered. We consider control options for BRD at different stages of the beef supply chain.

## Introduction

Antimicrobial^1^ resistance (AMR) constitutes an alarming public health threat, with a combined death toll estimated at about 50,000 lives a year in the United States and the European Union (1–3), and additional costs of treatment for infected patients up to USD 40,000. At present, there is considerable research demonstrating the impact of antimicrobial use (AMU) in animal agriculture on AMR in humans (4). Even if the overall quantitative impact of AMU in animal agriculture on public health remains difficult to assess (5), recent and growing awareness regarding AMR has driven governments to implement regulatory and voluntary public policies aimed at curbing AMU.

These, and intra-sectoral efforts to ensure antimicrobial stewardship, are all aimed at maintaining the antimicrobial susceptibility of pathogenic bacteria (6, 7). In the U.S., all label indications for antimicrobials (AM) used as growth promoters have been removed since December 31, 2016; meanwhile, other countries have implemented additional regulatory and voluntary measures to decrease AMU (8, 9).

Cattle production is the most important agricultural industry in the U.S., accounting in 2015 for $79 billion of the $377 billion of U.S. agricultural commodity cash receipts (10). Cattle may be afflicted by diseases, which affect the efficiency of the production process via different channels (11). First, diseases may decrease output, by increasing animal losses due to involuntary culling, mortality, thus decreasing the quantity of output sold. Second, diseases may decrease the efficiency of production factors, leading for example to an increase in the feeding period, or a decrease in feed conversion. Third, disease increases variable costs such as labor, prevention and treatment costs per unit produced. To limit the damages associated with diseases, farmers commonly use AM, as the major diseases afflicting beef cattle are infectious in nature. Aggregate data from the U.S. Food and Drug Administration (FDA) have shown that dairy and beef cattle accounted for ~50% of non-medically and medically important AMU in food animal production (12). In cow-calf operations, AM are mainly used to treat bovine respiratory disease (BRD), pinkeye, and digestive diseases (13). Feedlot operations use AM to prevent, control and treat BRD, which is by far the most frequently occurring disease, affecting up to 36% of cattle placed on feed (13). Infectious diseases may occur when favorable conditions allow pathogens to overwhelm the immune defenses of the calves, such as inadequate feeding, stress, high density of animals, detrimental weather or poor management (14). To control BRD, farmers may choose to prevent outbreaks, by improving their management practices with e.g., biosecurity measures or non-AM prophylaxis (vaccination) (15, 16). They may also choose to use AM either as prophylactic treatment before the occurrence of the disease, during an outbreak as pen-level metaphylaxis (control, per FDA), or as individual treatment of sick animals following the occurrence and diagnosis of the disease (17, 18). As the therapeutic efficacy of AM is high, their use clearly enhances overall animal productivity; as such, they remain a widely used tool, highly resistant to producers' budget constraints (14). Yet, it has been shown that good management practices can also decrease the morbidity and mortality of cattle; consequently, the quantity of antimicrobials required to raise fattened cattle (19, 20) appears reducible, suggesting that AM are at least partially substitutable with other production factors during the production process.

The U.S. beef system can be viewed as a supply chain from farm to table, starting with the production of calves in cow-calf operations and finishing with consumers of fresh or processed meat products. However, such an oversimplification of this system hides: (i) a tremendous variability of stakeholders present in any production cohort e.g., farmers, auction market, background and feedlot operators, retailers; and (ii) a high degree of heterogeneity of the economic agents e.g., very small to very large operations (21). Because of frequent changes in ownership, judicious management practices by cow-calf operators to limit BRD incidence and severity also benefit stakeholders further along the supply chain. This characteristic is the cornerstone of preconditioning programs, in which cow-calf operators implement specific measures to decrease BRD risk before weaned calves arrive at the feedlot. Calves at low risk of presenting with BRD should command a higher price from feedlot purchasers (22). If upon arrival at the feedlot calves are considered at high risk for developing BRD, current disease management practices consist of administering AM to decrease the likelihood of an outbreak of BRD occurring during the first weeks in the feedlot. Feedlot operators may routinely apply AM to all calves entering feedlots if it is costly (i.e., too risky) to identify and separate the low risk from the high risk calves; in fact, such a practice may itself further contribute to AMR.

A wide set of instruments are available for policymakers to mandate, direct or encourage reductions in AMU. Yet, some policies may perform better than others, depending on the context of their implementation. Ideally, any policy implementation should be preceded by an *ex ante* evaluation of its effectiveness and net benefit efficiency. Our objective is to provide a perspective on the benefits of various policy instruments aimed at reducing AMU in beef production. Because BRD is the most important infectious disease complex afflicting beef cattle and frequently necessitates AMU at several stages of the beef supply, we illustrate our research with the prevention, control and treatment of BRD. This paper is organized as follows: (i) first we describe the structural characteristics of the beef system, as well as farm operators' attitudes and motivations for farming, in order to identify potential bottlenecks limiting the effectiveness and efficiency of policies to be implemented, (ii) second, we identify potential market failures in the beef supply system, and (iii) lastly, we provide insights regarding the expected performance of an array of potential policy instruments aiming at curbing AMU in the beef production system.

## Structural Factors of Interest Affecting the Implementation of Policies Targeting AMU

### Overview of the Beef Industry

In 2017, 11.9 million tons of beef were produced in the U.S (23). This production is projected to increase in forthcoming years, driven by a stable domestic demand and an increase in exports (24). The beef industry is characterized by a set of consecutive activities such as breeding, feeding, and processing cattle. The beef industry involves multiple actors located throughout the U.S., even though only a few states contain the majority of the operations—mainly the Plain States^2^ (21). In the beef industry, cattle cycles also occur with an average length of 11.8 years, during which the number of beef cattle is expanded and then reduced, impacting the movement, placement, and processing of cattle (21).

**Figure 1:**
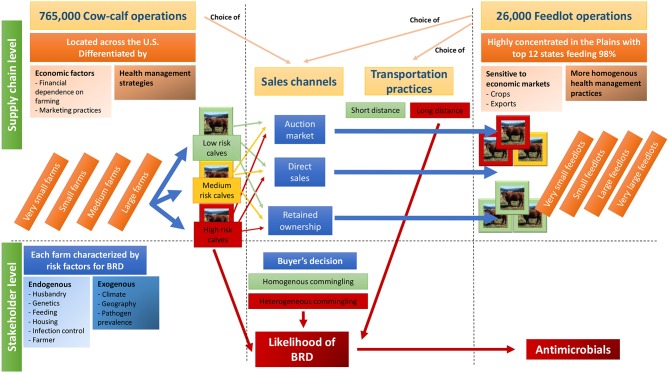
Framework of the beef production system as it relates to risk for bovine respiratory disease (BRD) and the need for antimicrobial use (AMU). Cow-calf operations of various size raise calves until weaning, which then reach feedlots via a sales channel and are processed on arrival. Depending on risk factors for BRD at the origin farm level, decisions made by the stakeholders of the beef supply network (shown in red in the figure), and the likelihood of BRD at the feedlot, AMU will be impacted in either a negative or positive direction.

The beef production system is heavily segmented. Seedstock purebred operations produce breeding animals with superior genetics; later, these are sold to commercial farmers and ranchers typically to cross-breed and raise calves for both production and breeding. Cow-calf operators produce and raise calves for up to a year after birth, when weaned calves weighing 180–320 kg are sold to stocker operators, backgrounders, or feedlots. Stockers purchase weaned calves at about 6 to 10 months old, and raise them on pasture until they gain 100-200 kg of extra weight over 3–8 months. Weaned calves may also be sold to backgrounding operations, where they are raised in dry lots. Feedlot operations finish calves, by feeding them high-energy rations combining forages and grains, to a slaughter weight of 450–550 kg. Some cow-calf or stocker producers may decide to keep their animals to produce grass-finished beef. Along this production process, many changes of ownership occur. Data estimating consumption of AM either in cow-calf or feedlot operations are scarce. It is noteworthy that the USDA has conducted a set of surveys providing information on the proportion of farms using AM (13, 25–27), but animal-level, farm-level and even production system-level AM consumption remain poorly known. Figure 1 gives an overview of the beef industry, from cow-calf operators to feedlot operators, emphasizing practices increasing AMU.

### Heterogeneity of Cow-Calf Operations

In 2018, approximately 32.5 million beef cows, which calved in the U.S. in 2018, were located on 725,000 beef farms (10, 28). Approximately 765,000 farms reported beef cow inventory. Most of these were small and part-time operations, and nearly 80 percent had fewer than 50 cows (29). Many smaller operations are hobby farms, in which income from off-farm sources greatly outweighs income derived from the farm. Even in larger operations, accounting for the largest part of U.S. production, cow-calf production is often not the primary enterprise (e.g., crops are the majority). This suggests that for some farmers, policies aiming to tax AMU or incentivize alternative practices might be inefficient, as economic considerations may not constitute the primary motivation for farming.

Cow-calf operations are not homogenous. The USDA ranks cow-calf operations into four categories depending on herd size (<50, 50–100, 100–200, >200) (30). Based only on this first level of classification, one readily can observe heterogeneity of operations regarding several components, such as motivations for farming, health management, or channels of sales. We regrouped the data extracted from these reports in Table 1. Though it is complicated to correlate the results of these reports, there seems to be an association between increased herd size and increased technology use (such as improved genetics), financial dependency and farming activity, and increased number of alliances. Alliances consist of two or more firms in the beef supply chain, which agree to cooperate for their mutual benefit, while remaining independent. As an example, they could agree to share information regarding health management (e.g., preconditioning programs).

**Table 1:** Characteristics of U.S. cow-calf operations [adapted from (21, 29, 30)].

**Variable**	**Subvariable**	**Unit**	**Herds**			
			**Small**		**Large**	
Herd size			<50	50–100	100–200	>200
National characteristics	% of U.S. beef operations	%	79.4	11	5.7	3.9
	% of U.S. beef cows	%	28.7	17.2	17.5	36.6
	% operator's work time	%	28.9	47.3	55.5	68.2
Motivations for farming	Primary income source	% of operations	5.3	24.1	42.8	65
	Supplemental income source	% of operations	78	68.3	50.9	31.7
	Other motivation	% of operations	16.7	7.6	6.3	3.3
Marketing	Conventional marketing	% of operations	60.5	68.7	68.4	67.8
	Organic marketing	% of operations	1.2	0.2	0.3	1.3
	Forward pricing	% of operations	2.3	3.1	6.9	15.4
Sales	Providing buyer information regarding health status	% of operations	28.2	43.4	57.5	74
	Selling to same people (vertical alliance)	% of operations	27.2	37.1	39.8	60.3
How diseases impact economics (% that agree)	Internal parasites	% of operations	49.6	57.1	69.4	63.7
	Scours	% of operations	58.8	65.2	76.6	76.4
	Shipping fever	% of operations	25.5	48.7	58	56.8
	Pinkeye	% of operations	30.4	46.8	48.7	48.6
Vaccination practices		% of operations	59.4	86.6	95.9	92.1
Against BRD	At least 1	% of operations	26.3	63.1	71.7	82
	Twice	% of operations	12.7	33.2	38.7	41.8
	More than 3	% of operations	0	8.2	6.7	16
Mortality	Born dead	% of animals	2.9	3	3.2	2.5
	Born alive and survived to weaning	% of animals	93.1	93	93.3	94.5
Weaning	Weaning weight heifers	kg	223	244	248	246
	Weaning weight bulls	kg	241	256	259	255
	Weaning age	days	201	207	207	209
	Weaning to sale period 0 day	days	56	44.8	27	34
	Weaning to sale period 1 to 30	days	15.4	19.9	21.2	12.4
	Weaning to sale period 30 to 60	days	12.2	12.8	16	28.4
	Weaning to sale period >120	days	9.8	8.2	9.1	20.4
Programs	BQA knowledge	% of operations	44.3	65.5	69.2	79
	BQA meeting attendance	% of operations	17.8	26.4	29.8	35.6
	Preconditioning program	% of operations	-	-	-	-

*BRD, Bovine Respiratory Disease; BQA, Beef Quality Assurance*.

### Complex and Changing Channels of Sales

After weaning, cow-calf managers may choose to retain ownership of their calves or contract with an investor prior to finishing (custom feeding) in a feedlot operation, or else sell the calves outright (Figure 1) (21). In large feedlots in the 2011 NAHMS Feedlot Report, custom feeding represented 30.7 vs. 40% of the cattle for feedlot sizes of 1,000 to 8,000, and >8,000, respectively (25). In the case of production contracts, the cow-calf operators retain ownership of animals throughout the growing process, thus allowing them to capture some of the extra value associated with high-quality animals, e.g., higher genetics or presenting low risks of contracting diseases, and access important production information (ideal for genetic selection) through the entire beef production chain.

Auction sales represent the largest share of transactions to source feeder cattle, varying from 27 to 47% of purchase in function of the feedlot size; direct sales account for 23 to 30% of the transactions (31). Changes in ownership and channels of sales may affect the traceability of the animals' health status, favor commingling and consequently enhance the spread of diseases. In addition, this raises the question of lack of symmetry of information, which may not allow for correct and efficient pricing of cattle as they move through the beef system.

### Concentration in the Feedlot Sector

In 2016, ~26,000 feedlot operations were identified in the U.S. Following arrival, calves are fed for a period varying from 100 to 250 days, largely dependent upon their weight at arrival, until they reach their slaughter weight (28). Cattle are mainly fed finishing rations (high-grain) prior to harvest, even though some operations background cattle by feeding them roughage rations prior to placing them on finishing rations.

The major differences between cow-calf operations and feedlots lie in their geographic location and their concentration. Because of the availability of feed grains and favorable climatic and geographic conditions, feedlot operations are concentrated in the Plains states, with the top 12 states feeding >98% of all beef cattle. Feedlots with <1,000 head of capacity account for more than 90% of U.S. feedlots. Feedlots with more than 1,000 head capacity feed 81% of all beef cattle. The top 10 feedlot companies have a combined capacity of 3.4 million head of cattle, out of a national total of 13.1 million. The high concentration in the feedlot sector could be perceived as an important and fairly straightforward driver to implement judicious practices of AMU, since it requires only a small number of large feedlots with good technical skills adopting judicious practices to drastically reduce the total number of animals treated with AM. In addition, such feedlots may be able to enforce BRD control practices in cow-calf operations (i.e., suppliers), while implementing contracts ensuring higher revenues to both buyers and sellers. On the other hand, because of high market concentration it is possible that a feedlot may not distribute to cow-calf operators the extra benefit of raising low-risk calves, decreasing the incentives for farmers to raise such animals.

As was the case for calf-cow operations, sorting feedlot operations by size helps identify variables for which feedlots exhibit different and relevant characteristics. Based on the most recent data available, we identify 3 variables—channels of sales, transportation of cattle from cow-calf operations to feedlot, and health management practices—for which we observe substantive differences according to feedlot size (<500, 500–1,000, 1,000–8,000, >8,000 heads) (see Table 2).

**Table 2:** Characteristics of U.S. feedlot operations [adapted from (21, 25, 28, 31)].

**Variable**	**Subvariable**	**Unit**	**Herds**			
			**Small**		**Large**	
Feedlot size			<500	500–1,000	1,000–8,000	>8000
Processing	Processing as a group	% of operations	80.4	91.7	98.2	99.9
	BRD vaccination	% of operations	64.5	91.7	92.9	94.7
	Clostridial vaccination	% of operations	55.9	72.2	76.6	72.5
	Injectable antibiotic	% of operations	35.3	47.8	39.1	71.5
	Implant	% of operations	35.1	56.2	72.6	85.6
	Parasiticide	% of operations	71.9	87.7	92.5	90.7
Conditioning	Preconditioning	% of operations	-	-	-	-
	No level of information at arrival	% of operations	-	-	8.4	4
	Little awareness of BQA	% of operations	49.5	24.3	8.1	1.4
Information	Absence of feedback from feedlot to supplier	% of operations	-	-	44.1	12.9
Transport	Source of shipment: auction	% of operations	36.1	62.5	64.5	67.6
	Source of shipment: other beef operation	% of operations	46.9	21.9	24.5	25.2
	Distance to feedlot (miles)	% of operations	92	237	319	394
Purchase (1)	Feedlots purchasing at auction	% of operations	25	62.3	62.4	68.6
	Feedlots purchasing via direct sale	% of operations	28.2	40.3	52.6	65.7
	Feedlots providing for custom feeding	% of operations	1.2	8.6	38	79.9
Purchase (2)	Calves purchased at auction	% of animals	41.4	46.8	37.9	27
	Calves purchased via direct sale	% of animals	23.1	23.9	26.5	30.2
	Calves for custom feeding	% of animals	2.6	7.8	30.9	40
	Calves born on feedlot operation	% of animals	32.5	21.4	-	-
Supplements in feed	Feedlot giving ionophores	% of operations	26.7	70.9	90.9	89.4
	Feedlot giving coccidiostats	% of operations	16.1	36.3	39.7	56.6
	Feedlot giving β-agonist	% of operations	3.5	11	29.1	55.9
Treatments records and practices	Training for medication	% of operations	-	-	80.8	97
	Written guidelines	% of operations	-	-	50.3	82
	Rectal temperature recorded	% of operations	-	-	52.2	79
	Date of treatment recorded	% of operations	-	-	84.4	98
Diseases	Death loss	% of animals	1.2	1.1	1.4	1.6

*BRD, Bovine Respiratory Disease; BQA, Beef Quality Assurance*.

### Time Component

Time is an important component to consider, as the cow reproduction cycle, as well as the time lag between calving and sale, lead to considerable delays between the time of decision making regarding management and the time the products are sold. Calving mainly occurs during winter and spring; in 2015, 72 percent of all calves in the U.S. were born in the first half of the year (10). Calves born in the spring are then weaned in the fall well after their digestive system can fully process whole feeds and once pastures start to decline with the impending winter months. This first characteristic of the calf supply affects the decision making of the farmers and other stakeholders of the beef system in three different ways: (i) congestion effects may be observed in the market, (ii) depending on the availability and price of commodities, cow-calf operators may decide to either keep or sell their calves, and (iii) depending on the region of origin, health disorders are more or less likely to occur during the calving season.

Second, unlike other animal production systems, beef production extends over a long production cycle, taking 1.5 to 3 years between birth and meat consumption. This suggests that delays between the onset of new practices and effects on AMU are to be expected. Additionally, the investments—in labor and in capital inputs—generally extend over several production cycles, and changes potentially required to decrease AMU may come at a high cost. This might create a dependence path, limiting the development of alternative options to AMU. Finally, budgetary constraints limiting farmers' investments might increase the sectoral antimicrobial demand.

### Controlling BRD in the Beef System: an Issue of Externality and Imperfect Information

Bovine respiratory disease complex is a multifactorial and multi-agent infectious disease. Debate has continued over the past 50 years as to whether BRD primarily exhibits features of a point-source or propagated (contagion) epidemic. By definition, BRD pathogens can be transmitted from one animal or farm, to another. However, most of the pathogens (both viral and bacterial) are known to present in otherwise healthy animals (i.e., are commensal or opportunistic pathogens). Control measures for BRD, through AMU or targeted management practices, create economic externalities. A first externality consists of AMU and the selection of resistant bacteria potentially damaging to the public health (32). Antimicrobials are used to kill pathogenic bacteria, but a side effect of any antimicrobial therapy is the selection of resistant bacteria in commensal flora, which thereafter may be transmitted to humans via direct contact, the food chain or the environment (5). As a public health threat, AMR generated by AMU in animal agriculture arguably reflects a market failure, thereby justifying the implementation of public policies. The policy instruments described below in this paper are aimed at curbing AMR, by encouraging a more optimal use of antimicrobials.

A second externality consists of the transmission of BRD pathogens across the supply chain, without the implementation of measures of prevention such as biosecurity measures, vaccination (33), or AMU. Pathogens can be transported from one site to another by calves—whether subclinically or clinically infected–and extend to other animals that come into contact with them (15). Because animals are often commingled at an auction market, poor management practices arising from only a fraction of farmers may generate high damages, borne largely by the buyer. Though the damages associated with BRD can be huge, the infectiousness of the pathogens is mild, and this externality is restricted to the beef system, therefore not justifying a public intervention. However, two reasons suggest the necessity to address this issue: (i) the costs to the beef supply system, and (ii) the fact that decreasing the number of calves at high risk of presenting BRD is likely to reduce AMU further down the beef supply, hence reducing at least partially the first externality described above.

It is reasonable to assume that cow-calf operators have relevant information regarding the health status of the calves they raise and sell, which they might also decide to hide from potential buyers. This asymmetric information dilemma can be approached under a principal-agent perspective. In this case, the buyer (principal) cannot observe the value of the animals to be purchased. As described by Hennessy and Wolf (33), adverse selection and moral hazard constitute two types of information problems in the principal–agent perspective. In the case of adverse selection, the agent has information that is relevant but unknown to the principal before the transaction, such as the health status of the calves. In such cases, the buyer is only willing to purchase calves at the more basic price paid for high-risk calves. This case is likely to happen frequently under the structure of the market described above. One solution to address adverse selection lies in the settlement of contracts in which the principal is able to distinguish low- from high-risk calves. In the case of moral hazard, the seller (agent) can implement control strategies to limit BRD occurrence that adds value to the animals purchased, but the principal cannot control their effective implementation. To address moral hazard, the principal may use incentives to share the risk related to the outcome, e.g., a variable remuneration linked to the growth performance of the calves after their transfer from cow-calf operation into feedlot.

Finally, any regulator has to face another type of incomplete information, arising from the ignorance of the amount of AM used at the farm level, and the actual damage associated with AMU in terms of AMR—both now and cumulatively into the future; though qualitatively well described, AMU remains complex to assess quantitatively. While controlling BRD in the beef system is likely to provide a benefit regarding AMR (and improve animal health and welfare), encouraging any non-AMU practices to reduce AMR have to be approached under uncertainty, which will affect the choice of instruments.

### The Choice of Appropriate Policy Instruments

The portfolio of potential policy instruments includes regulatory instruments e.g., AM bans, standards and certifications, and voluntary instruments such as economic incentives, agreements and industry self-regulation. The challenge consists of choosing the appropriate instrument(s), for which Bennear and Stavins (34) suggest three criteria: (i) the efficiency criterion (for our purpose, the ability of the instruments to reduce AMR while maximizing net benefits), (ii) the cost effectiveness criterion (reducing AMR and AMU at the lowest cost), and (iii) other economic and non-economic criteria.

**Figure 2:**
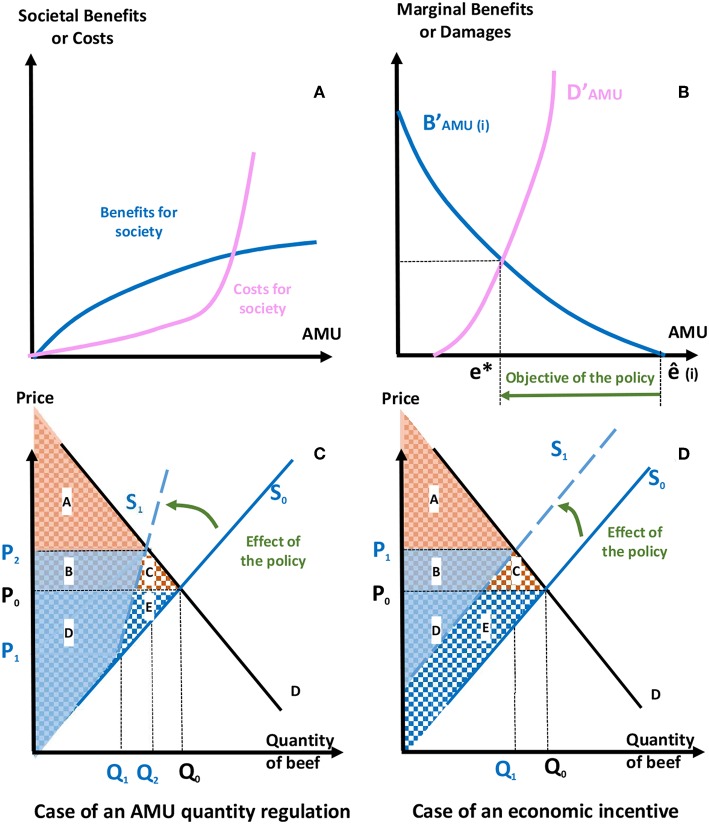
Graphical representation of the objectives and effects of public policies curbing antimicrobial use (AMU). **(A)** Society benefits (blue curve) from higher AMU from direct benefits for the farmers, but also from improved animal welfare, and safe and affordable food. The benefits are assumed to increase at a decreasing rate while in contrast, the costs for society, associated with antimicrobial resistance (AMR), are believed to increase at an ever-increasing rate (pink curve). **(B)** In the absence of regulation or alternative control, the societal optimal level of AMU is where the marginal benefit from the benefit curve in **(A)** is equated to the marginal cost from the cost curve in **(A)**, which is antimicrobials use at point ê_i._ A regulation aims at correcting the damage associated with AMU considering the marginal benefit and marginal cost. At the optimal level of AMU (e^*^), the marginal benefit to society equals the marginal damage (pink curve). The direct benefit to the beef producer, which is one component of the total benefit to society, is reflected in the beef supply curves in **(C,D)** where supply curves reflect the marginal cost of production. **(C)** Effects of a regulation controlling AMU quantity on the beef market. Antimicrobials are used by beef producers until the maximum quantity is reached, with similar production costs, and Q_1_ is produced. After this point, non-availability of AMU imposes higher production costs to producers, represented by the dotted blue line S'. The variation in producers' surplus PS is measured by the difference between the initial PS, (areas D+E, with blue squares), and the final PS (areas B+D, blue shaded). The variation in consumers' surplus CS is measured by the difference between the initial CS, (areas A+B+C, with orange squares), and the final CS (area A, orange shaded). **(D)** Effects of a tax on the beef market. A tax would lead to an increase in production costs, and the supply curve (S_0_) would shift up to (S'). Variations in consumer surplus (CS) and producer surplus (PS) read as for **(C)**.

Policies aim at sending a signal to producers, internalizing externalities into their production costs (Figures 2A,B). The choice of instruments to reach a target AMR reduction may change considering the specific abatement costs and enforcement costs (35). In human health, Coast et al. stated that the choice of policy options to decrease AMR should be done knowing the marginal abatement costs, which are likely to vary by practitioner, type of disease, and location (32). Additionally, because selection of resistant bacteria arises differently by bacteria species, AM classes, and treatment regimens, regulators should ideally take into account such biological parameters to determine best instruments. In practice, knowledge concerning the marginal cost of resistance is uncertain when it comes to implementing any policy regarding AMR, even though the options may acknowledge such variations. In the remainder of this section, we will present five categories of policy instruments: regulations, taxes, subsidies, tradable emission permits, and voluntary agreements. For each of these, we will provide existing examples (if any) of their use in curbing AMR. We will provide a perspective on their costs and their benefits in the U.S. beef sector, illustrated by variations in consumers and producers surpluses^3^ (see Figures 2C,D). We will present the advantages and limitations of each instrument, and will also describe the conditions required for their usage. This information is synthesized in Table 3.

**Table 3:** Characteristics of policy instruments applicable to decrease antimicrobial use (AMU) in beef production.

**Type of instruments**	**Objectives**	**Example**		**Advantages**	**Limitations**	**Requirements for use**
Regulations	Direct reduction of AMU	Set a target of AMU and control achievement	50% reduction target of AMU in farms animals in the Netherlands	Reduction objectives are set by the regulator allowing a quick achievement of the reduction	Less control of reduction achievements (time and amounts)	Monitoring of AMU
		Restrict use for selected AM classes	Specific requirements for the use of Critically Important Antimicrobials in France			
					Requires a significant level of information	
Economic incentives	Increase the cost of treating with AM, thus reducing AMU	Tax	Differential taxes on AM sales in Denmark	Requires little information on producer marginal costs	Economic incentives are less likely to abate AMR if this arises from a very few stakeholders consuming majority of AM	Estimating the societal cost of AMR
				Provides greater incentive to innovation		
Economic incentives	Encouraging alternatives treatment practices	Subsidy	-	No penalties for non-compliant producers	Specific public budget required	
Economic incentives	Decreasing the benefit of treating with AM	Tradeable permit	-	Market directs AMU to highest value	Transaction costs	Establishment of property rights
						Set a maximum amount to trade
Voluntary agreements	Decreasing the occurrence of diseases and non judicious use	Preconditioning program	Beef Quality Insurance in the U.S.	Improves animal health – benefit to feedlot operator	Farmer bears the upfront costs may not recoup expense	Correction of imperfect information
		Antimicrobial stewardship program				

*AM, antimicrobials; AMR, antimicrobial resistance*.

### Regulations

Several countries have already implemented regulations supervising the use of AMU under specific conditions, such as the ban for use of antibiotics in growth promotion in 2006 in the European Union and more recently for medically important antibiotics in the U.S. since January 1, 2017, and bans against therapeutic use for certain AM classes such as fluoroquinolones (8, 36, 37). Regulations would conceptually lead to an increase in production costs, and consequently an increase in meat prices. The economic consequences of antibiotic prohibition as growth promotors in cattle, pigs and poultry (38–41), of prohibition of all usage in dairy cattle (42, 43), and recently of prohibition of metaphylaxis in beef industry (44) have been evaluated. The authors of this last paper estimated a loss of $1.8 billion for the US beef industry associated with the ban of metaphylaxis. In these research reports, cost estimates were calculated by comparing potential changes from a single policy instrument with a business as usual scenario; assessments of other policies were not performed. Because the performance of all alternative policy instruments was not the purpose of these research reports, it is difficult to tell what the costs of alternative policy instruments would have been. However, research efforts examining pollutants have generally found that command and control approaches generally impose higher costs than other instruments (45).

A major advantage of regulations is that the regulator takes the responsibility to specify the reduction objective. This often leads to achieving the objective faster and with greater certainty. In the Netherlands, a 50% reduction target of AMU in farm animals was set in 2010, leading to a 56% reduction over the period 2007–2012 (46). In France, the regulation supervising the use of fluoroquinolones and 3rd and 4th generation cephalosporins implemented in 2016 led to a decrease in use of 87 and 94%, respectively, between 2013 and 2017 (47).

A drawback of regulations is that they come with higher administrative costs as well as the legal costs of enforcement. In the case of stronger regulations, another difficulty lies in the fact that some animals that need AM treatments would remain untreated, raising moral and ethical animal welfare concerns. To address this issue, alternatives exist of targeting only certain AM classes or practices of use. The performance evaluation of any such policy requires (1) documenting thoroughly the expected benefits associated with AMU related to their efficacy, and (2) estimating the benefits of restricting their use in specific cases, acknowledging that the selection of resistant bacteria follows complex longer-term patterns, even in the absence of antimicrobial use (5). Finally, imposing a uniform regulation is likely to penalize some producers more than others, depending on their baseline use of AMU, which often is a function of endogenous factors, but also exogenous factors, such as localization or disease prevalence.

### Economic Incentives

Taxes, subsidies, and cap-and-trade programs (tradeable permits) are common tools falling under the economic disincentive/incentive umbrella. Economic incentives provide rewards or penalties to the firms, to encourage a change in the use of resources. These have become popular in environmental policy; as one example, Harrington et al. (48) suggest that this gain in popularity over command and control tools may be explained by their expected higher efficiency, their adaptability, and a lower level of information required prior to implementation to reach a cost-effective pollution reduction. Indeed, the regulator does not need to know the shape of the abatement costs function for each producer. A condition to implement economic incentives is to observe the levels of emissions (analog is AM consumption), with either ideally AMR or AMU as a proxy. In the U.S., estimates of AM sales thus far exist only at the national level.

### Taxes

Some European countries have introduced taxes in their set of measures. In 2013, Denmark implemented differential taxes on antimicrobials by type. The tax rates varied between 0.8% for narrow-spectrum penicillins and other veterinary medicines, 5.5% for other veterinary antimicrobials and as high as 10.8% for critically important antimicrobials (49). Since then, a significant decrease in AMU has been observed; that said, taxes were just part of a wider set of measures, including regulations and risk communication campaigns, and it is therefore difficult to disentangle their separate effects.

Taxes have been identified both in human and animal health settings as a way to reduce AMR (32). Theoretically, setting a tax on antimicrobial sales, of an amount equal to the externality associated with AMU, would help correct for market failure (50). When producers choose their levels of inputs to production, they take into account their private marginal costs (Figure 2B, blue curve), while the marginal damages to society of the externality is overlooked in their decision process (Figure 2B, pink curve). In the absence of regulation, a beef producer (i) chooses to use antimicrobials up to the level ê_i_, at which point the cumulative benefits of use (blue curve) equal the cost. A tax implementation provides flexibility to producers, because they can determine their optimal level (e^*^) of AMU subject to the tax, and adapt their production process in reaction to the tax. However, determining the cost of the AMR externality is exceedingly complex, temporally distant, and difficult to estimate, which limits the efficiency of this option. In practice, taxes may be set at a level to simply target a fixed reduction. As for regulations, taxes would lead to an increase in production costs, shifting the supply to the left (Figure 2D).

### Subsidies and Tradeable Permits

To our knowledge, neither subsidies nor tradeable permits of AMU have been utilized, whether in human or veterinary medicine. Subsidies consist of encouraging producers to adopt virtuous practices. Conceptually, the mechanism of action is the opposite of taxes. Under the tradeable permit framework, the regulator sets a cap on the total amount of emissions (or use: in this analogous situation a reduction in AMU to a capped limit), and allocates permits to producers, who are allowed to buy or sell their permits. This enables producers facing high abatement costs to buy permits from those with lower abatement costs. Several factors need to be addressed prior to successful implementation, such as allocation of permits, localization issues, and types of use, probably limiting their potential use (32). In addition, implementing permits would require regulations regarding AM stewardship, ensuring that producers follow explicit rules regarding AMU, before trading.

It is obvious that regulatory measures and economic incentives could not be restricted to a single use of AM, such as the control of BRD, nor within a single animal sector. Instead, they would need to be targeted more broadly to AMU in beef production and other animal systems, meaning that an *ex ante* evaluation of their cost-effectiveness and efficiency would need to include analysis of other diseases. Overall, such measures would help to address the externality associated with AMU, but are unlikely to address information asymmetry across the beef supply and other commodities.

### Voluntary Agreements

Voluntary agreements are initiatives aimed at correcting a market failure, accepted by companies, or governmental or non-governmental organizations, in which participation is not legally binding (51). These have been increasingly used during the last 30 years as environmental policies, and more recently for public health policies (52).

One example in animal health, particularly relevant to our research, is the voluntary withdrawal of the previously approved labels for medically important antibiotics in food animals used for production purposes, implemented in 2013 by the FDA and finalized by December 31, 2016. This agreement was settled upon by the U.S. government and pharmaceutical companies delivering such products in the U.S. From a government point of view, the rationale for developing such agreements is that they are a cheaper and faster alternative for changing behaviors of stakeholders, compared to other enforced instruments. Businesses may join voluntary agreements to avoid regulations or the costs associated with enforcement, or to respond to pressures coming from lobbying groups or the public, as well as for marketing purposes. As one example, McDonald's announced in December 2017 a plan to reduce AMU in its beef suppliers by 2020 in Australia, Brazil, Canada, France, Germany, Ireland, New Zealand, Poland, U.K. and the U.S. (53). This follows a former plan implemented in poultry in 2015, which inspired many other food chains to follow suit. Whilst those kinds of agreements have proven their effectiveness, they may require important adaptations across many more operators in the far less vertically integrated beef system. Additional time before full adoption of practices is also required, as the supply chain is complex and the production cycles are long. Such commitment is likely to be followed by McDonald's competitors, and will place overall pressure on the entire beef system (both U.S. and globally) to reduce AMU. Remaining questions include how operators will react to AMU reduction targets, and which stakeholders will be better or worse off.

Another type of voluntary agreement, targeting more specifically BRD control, consists of preconditioning programs. This concept goes back many decades in North America, with varying degrees and eras of success. The concept of preconditioning relates to management methods surrounding the weaning period of beef calves, designed to decrease the stress and increase the immunity of calves and therefore reduce disease incidence at the feedlot (54). Several empirical research studies have found economic benefits to preconditioning (22, 55, 56); yet, the sustained adoption of preconditioning in the beef industry has been relatively low in North America (30, 57, 58).

Because preconditioning has been shown to decrease the incidence of BRD (19), encouraging such programs would likely indirectly curb AMU, constituting a win-win situation for both businesses and society. As described earlier, the strong heterogeneity observed in the cow-calf sector constitutes a limitation to the adoption on a large scale of good practices of BRD control. If cow-calf operators are not primarily financially motivated by their farming activity, then the constraints generated by implementing BRD prevention e.g., adaptations of infrastructures or extra labor, would not be offset by sufficient reward. In the ideal situation where cow-calf operators are willing to implement preconditioning, agreements should be designed in such a way that each stakeholder should accrue the benefits associated with the practice proportional to their input costs, with mechanisms designed to share the benefit across the beef supply. The fact that a small number of feedlot operations feed the majority of cattle constitutes an important asset to encourage preconditioning, as they have the capability to write and enforce a preconditioning agreement. The adoption of good practices by pioneering enterprises may also drive the adoption of similar measures by competitors. The result may be the emergence of two beef markets. The first would consist of large operations in which risk factors of diseases are controlled, certification of practices is available, and alliances are formed between the cow-calf and feedlot operators; in this market, agreements covering the goals, the outcomes, including monitoring and certification systems, and sanctions in case of non-compliance, would help correct information asymmetry. The second market would consist of smaller operations not willing to adopt preconditioning. Further research is needed to investigate how preconditioning could contribute to prevent AMR to better understand the potential reductions in externalities.

## Conclusion

In conclusion, the continued rise of AMR bacteria in animal and human health motivates the implementation of approaches to curb AMU in the beef sector. The public health threat and the high amounts of AM used in this specific sector constitute two factors justifying quickly addressing the issue of AMU in beef. In Europe, several countries such as the Netherlands, Denmark, Sweden, and France, have already implemented differential regulatory measures targeting specifically AM classes of clinical importance in human medicine, which successfully led to a quick reduction of their use, without decreasing the productivity of farmers. Yet, any policy targeting a very large panel of AM classes would likely penalize economic performances, as well as animal welfare, at least for a short period of time. Comprehensive empirical research is currently not available to help policymakers and stakeholders to evaluate meaningful measures that could be enacted in the beef sector. Designing a framework that models and assesses implementation of economic incentives such as subsidies, taxes, and tradeable AMU permits, would certainly produce information to help achieve reduction in AMR. Regardless of any implementation of these potential actions, the pressures exerted by consumers and retailers also have been shown to influence AMU in other production systems. Even though the complexity of the beef system may make achieving a reduction difficult, some progress has begun. Voluntary programs offer a cost-effective option allowing interested producers to improve their benefits and contribute to improve social welfare. Encouraging those types of programs by helping stakeholders cooperate in a very complex supply system has the potential to enable a substantial AMU reduction, and ultimately, work toward meaningful reductions in AMR. Overall, combining a set of instruments, spanning from really specific regulatory measures, to stewardship programs aiming at changing farmers' behaviors, is likely to maximize AMU reduction without decreasing farming profitability.

## Author Contributions

GL, YG, and HS contributed conception and design of the study. GL wrote the first draft of the manuscript. LT, KK, LV, HS, and YG wrote sections of the manuscript. All authors contributed to manuscript revision, read, and approved the submitted version.

### Conflict of Interest Statement

The authors declare that the research was conducted in the absence of any commercial or financial relationships that could be construed as a potential conflict of interest.

## Footnotes

^1^In this paper, we use “antibiotic” (active mainly against bacteria and derived from microbes) interchangeably with “antimicrobial”. Technically speaking, antibiotics are a subset of antimicrobials (a wider classification used to describe a broader range of chemicals with activity against viruses, bacteria, and other microbes).

^2^The Plains (or Great Plains) is an area covering about a third of the U.S., located in the states of Montana, North Dakota, South Dakota, Wyoming, Nebraska, Kansas, Colorado, Oklahoma, Texas, and New Mexico.

^3^The concept for consumer surplus is that consumers are willing to pay higher prices for quantities above the price line but need to only pay for these quantities at the equilibrium price, so that reduction in expenditures is a benefit (surplus) to them. In parallel, producers are willing to supply quantities at lower prices than the equilibrium price but they receive the higher price for their production, so that is a benefit (surplus) to them.

## References

[1] CDC. *Antibiotic Resistance Threats in the United States* 2013 (2013). Available online at:https://www.cdc.gov/drugresistance/pdf/ar-threats-2013-508.pdf (accessed July 12, 2019).

[2] ECDC The Bacterial Challenge: Time to react. Stockholm (2009). Available online at: https://ecdc.europa.eu/sites/portal/files/media/en/publications/Publications/0909_TER_The_Bacterial_Challenge_Time_to_React.pdf (accessed July 12, 2019).

[3] O'Neill J Tackling Drug-Resistant Infections Globally: Final Report and Recommendations. UK Government and Wellcome Trust (2016). Available online at: https://amr-review.org/sites/default/files/160518_Final%20paper_with%20cover.pdf

[4] Tang KL Caffrey NP Nóbrega DB Cork SC Ronksley PE Barkema HW. Restricting the use of antibiotics in food-producing animals and its associations with antibiotic resistance in food-producing animals and human beings : a systematic review and meta-analysis. Lancet Planet Heal. (2017) 1:316–327. 10.1016/S2542-5196(17)30141-9 29387833 PMC5785333

[5] Singer RS Williams-Nguyen J. Human health impacts of antibiotic use in agriculture: a push for improved causal inference. Curr Opin Microbiol. (2014) 19:1–8. 10.1016/j.mib.2014.05.014 24945599

[6] Van Boeckel TP Gandra S Ashok A Caudron Q Grenfell BT Levin SA Laxminarayan R. global antibiotic consumption 2000 to 2010: an analysis of national pharmaceutical sales data. Lancet Infect Dis. (2014) 14:742–750. 10.1016/S1473-3099(14)70780-7 25022435

[7] Van Boeckel TP Glennon EE Chen D Gilbert M Robinson TP Grenfell BT. Reducing antimicrobial use in food animals. Science. (2017) 357:1350–2. 10.1126/science.aao1495 28963240 PMC6510296

[8] US Food and Drug Administration Guidance for Industry. #213 New Animal Drugs and New Animal Drug Combination Products Administered in or on Medicated Feed or Drinking Water of Food- Producing Animals: Recommendations for Drug Sponsors for Voluntarily Aligning Product Use Conditions with. Fed Regist (2013). Available onlineat: https://www.fda.gov/downloads/AnimalVeterinary/GuidanceComplianceEnforcement/GuidanceforIndustry/UCM299624.pdf (accessed July 12, 2019).

[9] OIE OIE Annual Report on Antimicrobial Agents Intended for Use in Animals. Paris (2017). Available online at: http://www.oie.int/fileadmin/Home/eng/Our_scientific_expertise/docs/pdf/AMR/Annual_Report_AMR_2.pdf (accessed July 12, 2019).

[10] USDA Overview of the United States Cattle Industry. (2016). Available online at:usda.mannlib.cornell.edu/MannUsda/viewDocumentInfo.do?documentID = 1648 (accessed July 12, 2019).

[11] Mclnerney J Old economics for new problems -livestock disease: presidential address. J Agric Econ. (1996) 47:295–314. 10.1111/j.1477-9552.1996.tb00695.x

[12] FDA 2016 Summary report On Antimicrobials Sold or Distributed for Use in Food-Producing Animals. (2017). Available online at: https://www.fda.gov/…/UserFees/AnimalDrugUserFeeActADUFA/UCM588085.pdf (accessed July 12, 2019).

[13] United States Department of Agriculture Beef 2007 - 2008. Part I: Reference of Beef Cow-Calf Management Practices in the United States, 2007-2008. USDA Reports (2008) 1–89. Available online at: https://www.aphis.usda.gov/aphis/ourfocus/animalhealth/monitoring-and-surveillance/nahms/nahms_beef_cowcalf_studies (accessed July 12, 2019).

[14] Lhermie G Gröhn YT Raboisson D. Addressing antimicrobial resistance: an overview of priority actions to prevent suboptimal antimicrobial use in food-animal production. Front Microbiol. (2017) 7:1–11. 10.3389/fmicb.2016.02114 28111568 PMC5216048

[15] Fulton RW. Bovine respiratory disease research (1983-2009). Anim Heal Res Rev. (2009) 10:131–9. 10.1017/S146625230999017X 20003649

[16] Gorden PJ Plummer P. Control, management, and prevention of bovine respiratory disease in dairy calves and cows. Vet Clin North Am - Food Anim Pract. (2010) 26:243–59. 10.1016/j.cvfa.2010.03.004 20619182 PMC7135383

[17] O'Connor AM Coetzee JF da Silva N Wang C. A mixed treatment comparison meta-analysis of antibiotic treatments for bovine respiratory disease. Prev Vet. (2013) 110:77–87. 10.1016/j.prevetmed.2012.11.025 23276402

[18] DeDonder KD Apley MD. A review of the expected effects of antimicrobials in bovine respiratory disease treatment and control using outcomes from published randomized clinical trials with negative controls. Vet Clin North Am - Food Anim Pract. (2015) 31:97–111. 10.1016/j.cvfa.2014.11.003 25578389

[19] Duff GC Galyean ML. Board-invited review: recent advances in management of highly stressed, newly received feedlot cattle. J Anim Sci. (2007) 85:823–40. 10.2527/jas.2006-501 17085724 PMC7109667

[20] Hanzlicek GA Renter DR White BJ Wagner BA Dargatz DA Sanderson MW. Management practices associated with the rate of respiratory tract disease among preweaned beef calves in cow-calf operations in the United States. J Am Vet Med Assoc. (2013) 242:1271–8. 10.2460/javma.242.9.1271 23600786

[21] Field TG Beef Production and Management Decisions, 6th ed. New York, NY: Pearson (2018).

[22] Williams BR Devuyst EA Peel DS Raper KC The likelihood of positive returns from value-added calf management practices. J Agri Appl Econ. (2014) 1:125–38. 10.1017/S1074070800000675

[23] USDA ERS Livestock & Meat Domestic Data. (2018) Available online at: https://www.ers.usda.gov/data-products/livestock-meat-domestic-data/livestock-meat-domestic-data/#Beef (accessed July 12, 2019).

[24] Drouillard JS. Current situation and future trends for beef production in the United States of America - a review. Asian-Austr J Anim Sci. (2018) 31:1007–16. 10.5713/ajas.18.0428 29973030 PMC6039332

[25] USDA Feedlot 2011 Part III: Trends in Health and Management Practices on U.S. Feedlots, 1994–2011. (2013) Available online at: https://www.aphis.usda.gov/animal_health/nahms/feedlot/downloads/feedlot2011/Feed11_dr_Part%20III.pdf

[26] USDA Feedlot 2011 Feedlots With a Capacity of Fewer Than. (2013) Available at : https://www.aphis.usda.gov/animal_health/nahms/feedlot/downloads/feedlot2011/Feed11_dr_PartII.pdf (accessed July 12, 2019).

[27] Health UVS-C for E and A Types and Costs of Respiratory Disease Treatments in U.S. Feedlots. Fort Collins, CO: USDA (2013).

[28] National Cattlemen's Beef Association. Industry Statistics. (2018) Available online at: http://www.beefusa.org/beefindustrystatistics.aspx (accessed July 12, 2019).

[29] Mc Bride W Mathews K The Diverse Structure and Organization of U.S. Beef Cow-Calf Farms. (2011). Available online at: https://www.ers.usda.gov/publications/pub-details/?pubid=44532 (accessed July 12, 2019). 10.2139/ssrn.2114474

[30] USDA:APHIS:VS Beef 2007-08 Part I: Reference of Beef Cow-Calf Management Practices in the United States, 2007-08. United States Dept Agric (2009) N512-1008. Available online at: https://www.aphis.usda.gov/aphis/ourfocus/animalhealth/monitoring-and-surveillance/nahms/nahms_beef_cowcalf_studies (accessed July 12, 2019).

[31] USDA Feedlot 2011 Part I: Management Practices on U.S. Feedlots with a Capacity of 1,000 or More Head. (2013) Available online at:https://www.aphis.usda.gov/animal_health/nahms/feedlot/downloads/feedlot2011/Feed11_dr_PartI.pdf (accessed July 12, 2019).

[32] Coast J Smith RD Millar MR. An economic perspective on policy to reduce antimicrobial resistance. Soc Sci Med. (1998) 46:29–38. 10.1016/S0277-9536(97)00132-9 9464666

[33] Hennessy DA Wolf CA Asymmetric information, externalities and incentives in animal disease prevention and control. J Agric Econ. (2018) 69:226–42. 10.1111/1477-9552.12113

[34] Bennear LS Stavins RN Second-best theory and the use of multiple policy instruments. Environ Resour Econ. (2007) 37:111–29. 10.1007/s10640-007-9110-y

[35] Chávez CA Villena MG Stranlund JK The choice of policy instruments to control pollution under costly enforcment and incomplete information. J Appl Econ. (2009) 12:207–27. 10.1016/S1514-0326(09)60013-1

[36] European Union Regulation (EC) No 1831/2003 of the European Parliament and of the Council of 22 September 2003. (2003). Available online at: http://eur-lex.europa.eu/legal-content/PT/TXT/?uri = celex:32003R1831 (accessed July 12, 2019).

[37] Danish Ministry of Food and the Environment Vejledning Om Ordinering af Antibiotika Til Svin (Guidlines for Ordination of Antimicrobials to Pigs). (2018). Available online at: https://www.sst.dk/~/media/34F841A604D94FD596168CAC4F2D8A3D.ashx (accessed July 12, 2019).

[38] Sneeringer S MacDonald J Key N McBride W Mathews K Economics of Antibiotic Use in U.S. Livestock Production. (2015). Available online at: https://www.ers.usda.gov/publications/pub-details/?pubid=45488 (accessed July 12, 2019).

[39] Graham JP Boland JJ Silbergeld E. Growth promoting antibiotics in food animal production: an economic analysis. Public Health Rep. (2007) 122:79–87. 10.1177/003335490712200111 17236612 PMC1804117

[40] Brorsen BW Lehenbauer T Ji D Connor J Economic impacts of banning subtherapeutic use of antibiotics in swine production. J Agric Appl Econ. (2001) 3:489–500. 10.1017/S1074070800009263

[41] Mathews KH Jr Economic effects of a ban against antimicrobial drugs used in U.S. beef production. J Agric Appl Econ. (2002) 34:513–30. 10.1017/S1074070800009287

[42] Lhermie G Tauer LW Gröhn YT. The farm cost of decreasing antimicrobial use in dairy production. PLoS ONE. (2018) 13:e0194832. 10.1371/journal.pone.0194832 29566103 PMC5864045

[43] Lhermie G Tauer LW Gröhn YT. An assessment of the economic costs to the U.S. dairy market of antimicrobial use restrictions. Prev Vet Med. (2018) 160:63–7. 10.1016/j.prevetmed.2018.09.028 30388999

[44] Dennis EJ Schroeder TC Renter DG Pendell DL Value of arrival metaphylaxis in U.S. cattle industry. J Agric Resour Econ. (2018) 43:233–50. Available online at: http://www.waeaonline.org/UserFiles/file/JARE432_v1.pdf

[45] Tietenberg TH Emissions Trading : Principles and Practice, 2nd ed. Washington, DC: Resources for the Future (2006).

[46] Speksnijder DC Mevius DJ Bruschke CJM Wagenaar JA. Reduction of veterinary antimicrobial use in the Netherlands. The dutch success model. Zoon Publ Health. (2015) 62:79–87. 10.1111/zph.12167 25421382

[47] Anses Rapport Annuel Médicaments Vétérinaires Contenant Des Antibiotiques en France en 2017. (2018). Available online at: https://www.anses.fr/fr/system/files/ANMV-Ra-Antibiotiques2017.pdf (accessed July 12, 2019).

[48] Harrington W Morgenstern RD Choosing environmental policy : comparing instruments and outcomes in the United States and Europe. Resources for the Future, Washington, DC (2004).

[49] Høg BB Korsgaard H DANMAP 2016 - Use of Antimicrobial Agents and Occurrence of Antimicrobial Resistance in Bacteria From Food Animals, Food and Humans in Denmark. (2017). Available online at: https://orbit.dtu.dk/files/140535625/DANMAP_2016_LOW_241017.pdf (accessed July 12, 2019).

[50] Vågsholm I Höjgård S. Antimicrobial sensitivity — A natural resource to be protected by a Pigouvian tax? Prev Vet Med. (2010) 96:9–18. 10.1016/j.prevetmed.2010.05.003 20570379

[51] Karamanos P Voluntary environmental agreements: evolution and definition of a new environmental policy approach. J Environ Plan Manag. (2001) 44:67–84. 10.1080/09640560124364

[52] Bryden A Petticrew M Mays N Eastmure E Knai C. Voluntary agreements between government and business-A scoping review of the literature with specific reference to the Public Health Responsibility Deal. Health Pol. (2013) 110:186–97. 10.1016/j.healthpol.2013.02.009 23506799

[53] McDonald's Corporation Using our Scale for Good: McDonald's New Antibiotic Policy for Beef. (2018) Available online at: https://news.mcdonalds.com/stories/using-our-scale-for-good/antibiotic-policy-beef-2018 (accessed July 12, 2019).

[54] Thrift FA Thrift TA Review: update on preconditioning beef calves prior to sale by cow-calf producers. Prof Anim Sci. (2011) 27:73–82. 10.15232/S1080-7446(15)30452-6

[55] King ME Salman MD Wittum TE Odde KG Seeger JT Grotelueschen DM. Effect of certified health programs on the sale price of beef calves marketed through a livestock videotape auction service from 1995 through 2005. J Am Vet Med Assoc. (2006) 229:1389–400. 10.2460/javma.229.9.1389 17078803

[56] Roeber DL Umberger WJ The economic value of preconditioning programs in beef production systems. J Agric Resour Econ. (2002) 27:577.

[57] USDA NAHMS Beef 2007-08 Part III: *Changes in the U.S. Beef Cow-Calf Industry, 1993-2008*. (2009). Available online at: https://www.aphis.usda.gov/animal_health/nahms/beefcowcalf/downloads/beef0708/Beef0708_is_PartIII_Highlights.pdf (accessed July 12, 2019).

[58] Canfax Research Services Economic Considerations on Preconditioning Calves. (2015) 1–8. Available at: http://www.canfax.ca/Samples/Preconditioning%20Sept%202015.pdf (accessed July 12, 2019).

